# Evaluation of Event-Based Corner Detectors

**DOI:** 10.3390/jimaging7020025

**Published:** 2021-02-03

**Authors:** Özgün Yılmaz, Camille Simon-Chane, Aymeric Histace

**Affiliations:** ETIS UMR 8051, CY Paris Cergy University, ENSEA, CNRS, F95000 Cergy, France; camille.simon-chane@ensea.fr (C.S.-C.); aymeric.histace@ensea.fr (A.H.)

**Keywords:** event-based camera, corner detector, event-based corners

## Abstract

Bio-inspired Event-Based (EB) cameras are a promising new technology that outperforms standard frame-based cameras in extreme lighted and fast moving scenes. Already, a number of EB corner detection techniques have been developed; however, the performance of these EB corner detectors has only been evaluated based on a few author-selected criteria rather than on a unified common basis, as proposed here. Moreover, their experimental conditions are mainly limited to less interesting operational regions of the EB camera (on which frame-based cameras can also operate), and some of the criteria, by definition, could not distinguish if the detector had any systematic bias. In this paper, we evaluate five of the seven existing EB corner detectors on a public dataset including extreme illumination conditions that have not been investigated before. Moreover, this evaluation is the first of its kind in terms of analysing not only such a high number of detectors, but also applying a unified procedure for all. Contrary to previous assessments, we employed both the intensity and trajectory information within the public dataset rather than only one of them. We show that a rigorous comparison among EB detectors can be performed without tedious manual labelling and even with challenging acquisition conditions. This study thus proposes the first standard unified EB corner evaluation procedure, which will enable better understanding of the underlying mechanisms of EB cameras and can therefore lead to more efficient EB corner detection techniques.

## 1. Introduction

Event-Based (EB) cameras, a promising new technology, consist of independent pixels that acquire data only if there is a change in their field of view. As such, the amount of information that must be processed, particularly in surveillance tasks, is greatly reduced. Each pixel operates asynchronously; therefore, the sensor can achieve low latency in its output and can spot fast variations in the scene.

By providing high temporal resolution and high dynamic range, event-based cameras are revolutionising the way machines perceive the visual world. Accordingly, commercial interest [1,2] has quickly increased in order to exploit the intrinsic advantages over traditional visual cameras for a variety of applications such as automotive, augmented reality, surveillance, and monitoring [3]. In particular, EB sensors have been increasingly explored for navigation and have been creating much excitement, as well as hope that they can push new boundaries [4,5,6]. That being said, their asynchronous nature has imposed a paradigm shift in the way perceptual information is acquired and processed. Hence, it has been necessary to redefine existing image processing tools in an EB formulation.

Image corners are distinct features that can be located with high precision, therefore making them desirable for especially fine localisation and tracking solutions. With a similar motivation, a few EB corner detectors [7,8,9,10,11,12,13] have been developed aiming at the precise localisation at high speed and in High Dynamic Range (HDR) conditions. For a high level understanding, a brief overview of these EB corner detectors is given in Table 1. More details on the selected detectors are given in Section 2.2. Unsurprisingly, except the plane fitting method [7], which searches the space-time representation of the event stream for the intersection of moving edges, most of these methods can be also considered to be an EB adaptation of well-known image-based techniques such as the Harris [14] and FAST [15] corner detectors. Often, each polarity is treated separately, and algorithms are tested on fast moving scenes.

Unfortunately, most of the experiments have not evaluated detector performances in HDR conditions even though the advantages of EB cameras in HDR are discussed in the papers. Moreover, new evaluation procedures are introduced every time a new algorithm is proposed perhaps to distinguish their solutions in a favourable manner. Some evaluation criteria [10,13] only rely on intensity images, which are limited to less interesting conditions (i.e., moderate motion speed and illumination conditions), while others [9] associate the nearby corners without considering the actual feature trajectory. Among all, the triangulation-based evaluation criterion [12] is found to be the most rigorous method that can measure the performance of the detectors also in challenging scenarios. However, this evaluation protocol completely puts aside the comparison of EB corners with image corner detectors, which could be still insightful to understand the underlying processes of the EB techniques as the ultimate goal is to keep the existing features and complements in challenging scenarios.

In this paper, we present a unified EB corner detector evaluation protocol that can provide better understanding of the underlying processes of EB cameras. We combine the strengths of the evaluation procedures proposed by the previous EB corner detector papers with our additional evaluation metrics. The primary part of the evaluation investigates the performance in terms of computational complexity and precision by using the camera trajectory, while the secondary part provides the accuracy metrics by comparing these with the intensity image information. Our comprehensive evaluation protocol is used to assess the performance of five EB corner detectors [8,9,10,11,13] in various challenging scenarios, including HDR conditions, which is has been barely investigated until now, using a public dataset. As such, our comprehensive evaluation methodology already shows its significance by indicating research avenues to explore and infertile research directions to avoid based on the current state-of-the-art. The contributions of this paper are thus two-fold: the first unified EB corner detection evaluation protocol and the thorough comparison of existing EB corner detectors using this protocol.

The paper is structured as follows: Section 2.1 introduces the necessary EB terminology used in the paper. Section 2.2 describes the EB corner detectors that are evaluated. Section 2.3 details the elements of the proposed evaluation criteria. The results of each evaluation criterion are given in Section 3 and discussed in Section 4, while the conclusions are summarised in Section 5.

## 2. Materials and Methods

### 2.1. Event-Based Computing Terminology

EB cameras output a stream of asynchronous events where each event $e_{i}$ describes the contrast change at a given pixel location $\left(x_{i},y_{i}\right)$ and at a given time $t_{i}$. This representation can be described as:

(1) $$e_{i}=\left(t_{i},x_{i},y_{i},p_{i}\right)=\left(t_{i},u_{i},p_{i}\right)$$

where $u_{i}$ is the pixel location and $p_{i}$ is the polarity describing the brightness change as positive (i.e., increasing intensity) or negative (i.e., decreasing intensity) over time.

#### Surface of Active Events

**Figure 1:**
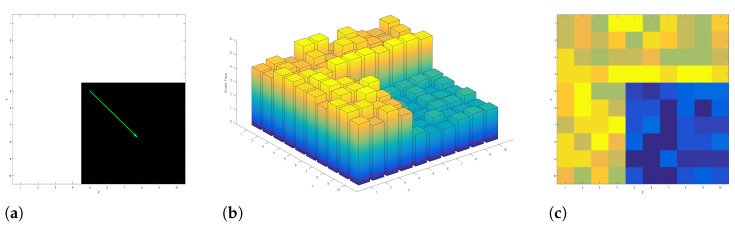
Surface of Active Event (SAE) representation of positive events. (**a**) The intensity scene where a black rectangle object is moving towards the green arrow; SAE representation of the resulting positive events in (**b**) 3D and (**c**) 2D (i.e., the time information is colour coded).

The Surface of Active Event (SAE) is a concept that describes a time-space representation of events [16]. An SAE stores the latest event trigger time (i.e., time dimension) of a pixel (i.e., spatial dimension). Hence, the SAE is defined for the full sensor dimension and updated by each event at its pixel location. However, event polarity information is not explicitly embedded. Generally, events of different polarity form separate SAEs. For a black rectangle object moving towards the right bottom corner of a scene, Figure 1a–c shows the resulting SAE of positive polarity events in 3D and 2D, respectively.

### 2.2. Event-Based Corner Detectors

This study focuses on five EB corner detectors for their performance evaluation: the event-based Harris corner detector (eHarris) (using the public implementation provided by the authors of eFAST [9] https://github.com/uzh-rpg/rpg_corner_events) [8], eFAST (using the public implementation provided by the authors of eFAST [9] https://github.com/uzh-rpg/rpg_corner_events) [9], Arc (using the public implementation provided by the authors https://github.com/ialzugaray/arc_star_ros) [10], Fast and Asynchronous (FA)-Harris (using the public implementation provided by the authors https://github.com/ruoxianglee/fa_harris) [13], and the Continuous Harris Event Corner detector (CHEC) (using the original implementation of the authors) [11]. In this evaluation, the LocalPlane [7] and SITC [12] algorithms are not included. In the case of LocalPlane [7], the reasoning is its high computational complexity (i.e., multiple optimisation process per event), which is considered less attractive for EB applications [9]. In the case of the SITC [12] algorithm, neither the trained model nor the fine details of the training procedure are available in order to achieve the required confidence level for this comparative analysis. Moreover, the publicly available dataset is missing important intensity information for the algorithm training to replicate the results accurately.

The algorithms were all implemented in C++, using the Robotic Operating System (ROS) and OpenCV libraries. This section provides a brief description of each method.

#### 2.2.1. Event-Based Harris Corner Detector

The event-based Harris corner detector [8] (eHarris) is an event-based adaptation of the Harris detector [14]. For each polarity, the algorithm establishes the SAEs and then binarises these SAEs by thresholding the newest *N* events. By doing so, the 3D SAE information is converted to a 2D binary frame where 0 and 1 represent the absence and presence of an event in a given pixel location. If we let $\sum_{b}$ as the local patch of the binary SAE, which is centred at the pixel location of the newest event e, the symmetric Harris matrix *M* can then be computed as:

(2) $$M=\sum_{e\in \sum_{b}}g\left(e\right)\nabla I\left(e\right)\nabla I^{T}\left(e\right)$$

where g(e) is a Gaussian window function and ∇I(e) is the gradient of the binary image patch $\sum_{b}$ convolved with a Sobel filter. Like the Harris detector, the corner metric *H* of eHarris is given by:

(3) $$H=\det\left(M\right)-k\;\cdot \;\mathrm{Tr}{\left(M\right)}^{2}$$

where k=0.04 is an empirically defined parameter. If *H* is larger than a certain threshold, the newest event e is classified as a corner. In this study, we use the improved version of eHarris by [9], which defines the newest *N* events for each local patch of the SAE rather than the global SAE. Thanks to this spatially-adaptive solution, the new version of the algorithm is scene and sensor size independent; therefore, the evaluation can be done with the best possible performance.

The challenges of this algorithm are: (1) the selection of *N* number of events, which is a hand-tuned parameter depending on the scene complexity, (2) for each event e, a frame-like binary patch is processed, which causes time delays due to number of time-consuming computations (e.g., convolution).

#### 2.2.2. Event-Based FAST Corner Detector

**Figure 2:**
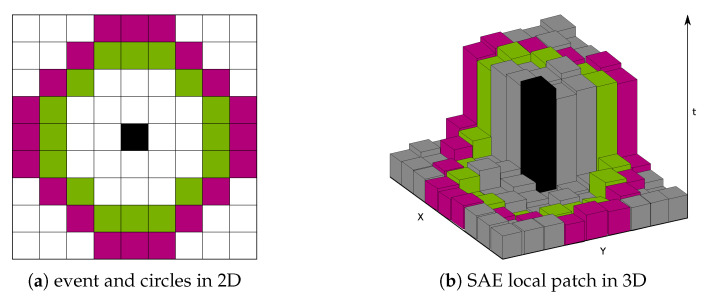
The inner (green) and outer (purple) circles that are centred at the current event (black) for given local SAEs. In this example, the event under consideration (black) is classified as a corner event.

The event-based FAST corner detector [9] (eFAST) is an event-based adaptation of the FAST detector [15]. For each polarity, the algorithm establishes an SAE and extracts a local SAE patch that is centred at event e, like in eHarris. Within this window, the algorithm searches for continuous streaks on two different circles, which are referred as the inner and outer circles, as shown in Figure 2. If the search finds a continuous streak that has a length equal to the third of each circle for a given patch, then the event e is classified as a corner event.

#### 2.2.3. Asynchronous Corner Detector: Arc

The asynchronous corner detector, called Arc [10], can be considered as a modified version of the eFAST detector [9]. In addition to the corner definition of eFAST, Arc also searches for corners with angles greater than $180^{\circ }$. This is achieved by searching the arc as in eFAST, but this time in both directions: clockwise and counter-clockwise.

Instead of the traditional SAE, Arc uses a modified version of the SAE, which stores the additional information of reference time $t_{r}$ in addition to the spatiotemporal structure (i.e., event position and time) of traditional SAEs. While the spatiotemporal information of the SAE is updated with each event, the related $t_{r}$ is only updated with the latest event time: (1) if the previous activation of the same pixel is older than a predefined time-window κ, which is set to 50 ms during the experiments [10]; (2) if the polarity of the previous event of the same pixel differs. By doing so, the reference time $t_{r}$ provides a sort of noise filtering to traditional SAEs caused by the hardware when consecutive activation of the same pixel is faster than the timestamp registration process [10].

The algorithm uses this new filtered SAE with the reference time $t_{r}$ in order to form the required spatiotemporal space, which is called SAE* hereinafter. Similar to [9], a circular mask of a 3 and 4 pixel radius centred at the current event location within the local SAE* is searched for continuous streaks (i.e., the oldest timestamp of the streak is newer than the adjacent locations on the circle) with the same lengths (i.e., 3–6 pixels for the inner and 4–8 pixels for the outer circle), as in [9]. The difference of Arc lies in the fact that the complementary streak on the circle is also checked for whether the length condition is satisfied or not (i.e., by the double directional search). By checking the complementary streak, Arc includes the corners with angles greater than $180^{\circ }$.

#### 2.2.4. Continuous Harris Event Corners

The Continuous Harris Event Corner detector (CHEC) [11], is based on the convolution of the event stream as the internal state of a continuous-time filtering technique. By employing a high-pass filter on the system, the drifting sensor noise over time, which shows low temporal frequency, is removed. As an internal state of the filter, CHEC establishes the image gradients ${\hat{G}}_{x}\left(x,t\right)$ and ${\hat{G}}_{y}\left(x,t\right)$, which are used to compute the Harris corner metric [14].

Contrary to eHarris, the image gradients ${\hat{G}}_{x}\left(x,t\right)$ and ${\hat{G}}_{y}\left(x,t\right)$ are related to the intensity information of the scene rather than an artificially created binary image from the SAE by thresholding for each polarity. Instead of convolving the intensity image with a 3×3 Sobel kernel, each event is converted to nine artificial kernel events where the kernel is centred at the original event. These artificial events have the same timestamp as the original event and perform an exponential decay at pixel locations to estimate a priori gradient $\hat{G}\left(x,t_{i}^{-}\right)$ at given event location x.

(4) $$\hat{G}\left(x,t_{i}^{-}\right)=e^{-\alpha (t_{i}-t_{x})}\hat{G}\left(x,t_{x}\right)$$

where $t_{i}$ is the event timestamp, α is the cut-off frequency to reduce the low temporal noise, and $t_{x}$ is the previous timestamp at pixel x. Then, a posteriori gradient $\hat{G}\left(x,t_{i}^{+}\right)$ is estimated as:

(5) $$\hat{G}\left(x,t_{i}^{+}\right)=\hat{G}\left(x,t_{i}^{-}\right)+p_{i}c\left(K*\delta_{x_{i}}\right)\left(x\right)$$

where $p_{i}$ is the polarity, *c* is the EB camera contrast threshold, *K* is the kernel, and $\delta_{x_{i}}$ is the delta function that is non-zero at event timestamp $t_{i}$ and location x.

By using this gradient estimation, an internal state that describes the intensity Harris corner metric is calculated locally. It is important to note that non-maximum suppression techniques are necessary to retrieve decent corners from this internal state. This non-maximum suppression is applied periodically by the authors for the results that were presented in their paper [11]. In this context, CHEC can be considered as a hybrid solution since the first part of the computations that computes the corner metric is EB, while the detection of a decent corner by employing the non-maximum suppression technique is a somewhat frame-based approach.

Our study focuses on the EB performance of the algorithm; therefore, we do not implement a frame-based non-maximum suppression technique, which would greatly increase the computation time. A localised non-maximum suppression solution that could be implemented in EB is found to extremely penalise the corner event stream and presents frequent discontinuities, which is not favourable. Instead, a very high corner threshold (i.e., 0.015) is employed such that the detector outputs a comparable event corner stream for the evaluation.

#### 2.2.5. Fast and Asynchronous Corner Detector

The Fast and Asynchronous corner detector (the so-called FA-Harris detector) [13] is a corner detection pipeline that uses features from Arc [10] and eHarris [9]. First, the event stream is filtered before forming the SAE, and the corner events are selected as in [10]. The difference is that these detected EB corners are considered as candidate events in the FA-Harris algorithm. Consecutively, these events are reprocessed by eHarris [8] in order to be selected as the real EB corners, which is claimed to be a more refined output.

### 2.3. Evaluation Criteria

#### 2.3.1. Reduction Rate

Considering the high output rate of the EB camera, reducing the event-stream into the most relevant and manageable number of events results in the need for feature extraction. In this study, the reduction rate $R_{\left(\%\right)}$ is defined as:

(6) $$R_{\left(\%\right)}=\left(1-\frac{\#e_{corner}}{\#e_{total}}\right)\times 100$$

where $\#e_{corner}$ is the number of corner events and $\#e_{total}$ is the total number of events as described in previous studies [9,10].

#### 2.3.2. True Positive Rate/Sensitivity

Similar to the methods presented in [10,13], intensity image corners detected by the Harris detector [14] are tracked by using KLT [17] over time when compatible with the dataset (i.e., no motion blur, favourable illumination conditions).

For each track *i*, a pair of successive image corners is connected by a line segment, which forms the centre of an oblique cylinder with radius r pixels, and the depth is the time difference between their respective images. All events within this cylinder are considered as the true corner events generated by the actual image corners, and the total number of these events is defined as $E{\left[\leq r\right]}_{i}^{All}$. For each EB detector, the detected event corners within this cylinder are labelled as the True Positives (TPs) and defined as $E{\left[\leq r\right]}_{i}^{Det}$. On the other hand, events that are within the cylinder, but not detected by the EB detector are considered as the False Negatives (FNs). Consecutively, the True Positive Rate (TPR) of each EB detector can be described as in Equation (7), which allows us to evaluate their sensitivity.

(7) $$TPR=\frac{TP}{TP+FN}=\frac{\sum_{i}E{\left[\leq r\right]}_{i}^{Det}}{\sum_{i}E{\left[\leq r\right]}_{i}^{All}}$$

Contrary to earlier studies [10,13], we do not define another cylinder with a larger radius, which is used to identify the negative samples (i.e., detected events that are not actual event corners). Such a metric is considered as possibly misleading for our deductions since the underlying processes of image and event generation are different and EB detectors are likely to be more sensitive than intensity-based imagers. While all image corners are expected to be detected by an ideal EB corner detector, the reverse is not necessarily true.

Since our end goal is to understand the relationship between the image and EB corners, cylinders with three different radius values (i.e., 1.5, 3.5, 5 pixels) are analysed in this study. In previous studies, only one radius (i.e., 3.5 pixel) was used [10,13].

#### 2.3.3. 3D Reprojection Error

Considering the fact that EB cameras have higher output rates than frame-based cameras, a stable EB corner detector should provide continuous tracks without requiring complex tracking techniques. Therefore, decent tracking results can be achieved by employing a simple nearest neighbour matching in space and time, as discussed by [12], which we also use in our evaluations.

For tracking, each new coming event at time $t_{i}$ is compared with the feature tracks that are updated within the last δT seconds and within the radius of *r* to the event. Then, the event is assigned to the longest track within this list. In order to remove the noise from the feature tracks and increase the feature location accuracy, the tracker averages the location of the last *n* events as the location of the feature and does not evaluate the location of the first *n* events. The details of this simple nearest neighbour matching algorithm can be found in [12]. For our evaluations, *r*, δT, and *n* are taken as 3 pixels, 50 ms, and 10 events, respectively.

In order to evaluate the performance of EB corner trackers using public datasets that are non-planar and/or have an event rate higher than the ground truth rate, we used the reprojection error associated with the 3D triangulation of events belonging to each feature track (described in the previous paragraph) for a given ground truth camera pose, as in [12,18,19]. As in [12,18], tracks with a reprojection error of less than 5 pixels are considered as “valid tracks”. The quality of the EB corner detector is defined by the percentage of valid tracks and the mean reprojection error of the triangulated 3D points over the respective valid tracks. Moreover, the average track lifetime per dataset is also provided to evaluate the traceability of the EB corner detectors.

## 3. Results

### 3.1. Datasets

**Figure 3:**
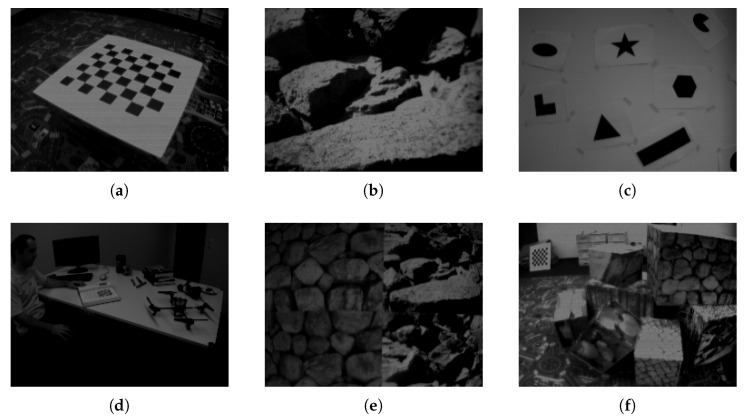
Dataset scenes: (**a**) chequerboard, (**b**) slider, (**c**) shapes, (**d**) dynamic, (**e**) poster, (**f**) boxes.

The evaluation of EB corner detectors is performed by using the public DAVIS 240Cdataset. A few scene extracts are shown in Figure 3, and further details can be found in [20]. Each dataset consists of DAVIS images, events, camera calibration, and the ground truth information. The ground truth for the slider is simply given by its position. The ground truths for all the other datasets are recorded using a motion capture system. All scenarios last around 60 s with the exception of the slider dataset, which is around six seconds.

Compared to the previous studies [9,10,13], our study also analyses, for the first time, the following scenarios: calibration, slider_close, slider_far, slider_hdr_close, slider_hdr_far, and hdr_poster. Among these datasets, the suffixes “close” and “far” describe the distance to the planar scene, whereas “hdr” describes the challenging illumination conditions. A high total number of events indicates more complex and textured scenes. Except for the slider dataset, the duration of all acquisitions is similar; therefore, a significant increase in the number of events can be related to the scene textures or dramatic changes in scene illumination.

### 3.2. Reduction Rate Performance

For the entire dataset of this study, the event reduction rate for each EB corner detector is shown in Table 2. For relevant scenarios, our results are compatible with the results of the previous studies [9,10,13]. There is only a small bias of approximately 0.3% with the FA-Harris detector with its original paper even though the authors’ public implementation was used. This could be due to the corner threshold selection, which was not given by their paper (our study sets the corner threshold to eight).

For all detectors, scenarios with “shape” scenes have the lowest reduction rate compared to the rest of the scenarios. This can be related to the low texture of the scene where events are generated by geometrical shapes on a white background. Extreme illumination (spotlight) that is present at the second half of the slider_hdr datasets results in a decrease in the total number of events. This means that even though EB cameras can operate in extreme illumination conditions, without parameter adjustments, their sensitivity degrades. However, there is no significant effect of scene illumination on the reduction rate of EB corner detectors.

Moreover, the reduction rate of EB corner detectors depends on the scene, but not on the camera trajectory (i.e., the reduction rate for translations, rotation, and six degree of freedom trajectories of the same scene are similar for all detectors). For the slider dataset, which is the slowest among all and has the only constant speed trajectory, CHEC seems unable to detecta tangible number of EB corners. This could be related to a lower number of updates of the internal gradient estimates due to less events generated by the slow motion and eventually leading to a lower Harris corner metric, which remains below the selected constant threshold.

In Table 2, the highest reduction rate for each scene is highlighted. However, CHEC is not the best EB corner detector. The purpose of this metric is to evaluate the data reduction of EB corner detectors, as this is interesting for higher level algorithms. This metric by itself cannot assess if the remaining feature events are all of the intended data or good enough for post-processing. The higher reduction rate is favourable as long as all meaningful data are kept. Therefore, to make more comprehensive conclusions, this metric shall be used along with the other metrics. Hence, this fact again suggests the importance of our comprehensive evaluation procedure rather than single aspect studies that may be misleading.

### 3.3. True Positive Rate/Sensitivity Performance

In our analysis, we used the first 10 s of each dataset and only the image corner tracks that were longer than one second. Table 3 shows the TPR of the event corner detectors with respect to the tracked intensity corners for different radii. As expected, the TPRs of eHarris and eFAST for a radius of 3.5 pixel were found to be similar to the results given in [10]. Small variations compared to [10] are associated with the definitions of track refinement and short tracks, which were not explicitly described in the original paper.

The analysis of the different trajectories of the same scene suggests that the TPRs of EB corner detectors are more closely related to the scene structure than the camera trajectory. EB detectors perform particularly well for highly geometric scenes with strong gradients such as the calibration and shapes datasets. This could be related to the constant contrast threshold of the EB camera over different dataset acquisitions. For the shapes dataset in particular, all EB detectors provided high precision, which can be identified by a significant decrease in TPR from 1.5 pixels to five pixels, as shown in Table 3.

The CHEC detector shows a significant degradation of its performance for the slider dataset, which has the slowest trajectory among all the datasets. This could be related to the fewer updates at the internal gradient estimates due to the fewer events generated by the slow motion and eventually leading to a lower Harris corner metric, which remains below the selected constant threshold.

FA-Harris shows the poorest performance among all EB corner detectors, except for the slider dataset. This result is interesting because FA-Harris claims to achieve better performance by combining the strengths of eHarris and Arc. However, our findings show that such a combination rather penalises the TPR of the detector. On the other hand, eHarris shows one of the best TPRs among all EB corner detectors over all datasets. Since the TPR is based on the tracks of image-based Harris corners and eHarris uses the same algorithm on artificially created SAE images, this result may indicate a close relationship between the SAE and intensity images. However, such reasoning requires further analysis in order to be justified.

**Figure 4:**
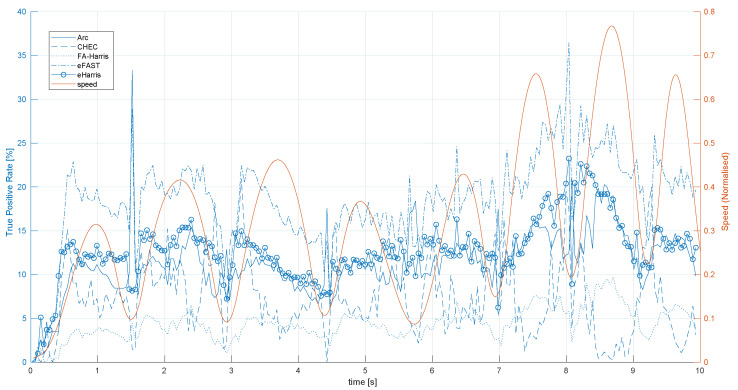
TPR of EB corner detectors over time for the shapes_translation dataset along with the normalised speed of the trajectory.

Figure 4 shows the TPR of EB corner detectors over time for the shapes_translation dataset along with the normalised speed of the trajectory, which is also proportional to the number of events per second. As can be seen for all EB detectors, the initial TPR is zero and requires about 30 ms to reach its average value over the entire evaluation duration. This shows that none of the EB corner detectors reach their nominal performances immediately after the acquisition start. This is as expected because all algorithms somehow accumulate events in the form of either SAEs or internal gradients. Without sufficient accumulation of event information over time, their algorithm cannot detect them.

At the times of directional changes in motion, as shown by the minima of speed in Figure 4, the number of event fired decreases significantly compared to fast motion. These are also the times when EB corner detectors’ TPR decreases compared to high speed trajectories. Nevertheless, Arc shows comparatively better results for directional changes in motion. This could be related to the fact that Arc is specifically designed to detect corners with angles larger than $180^{\circ }$, and directional motion change may result in small corner angles becoming larger from the EB perspective. Moreover, we observe a general trend of better TPRs at increasing speeds, which can be seen at the second half of the trajectory for eFAST, eHarris, and Arc. This could be related to the fact that a higher speed generates more events and consecutively more updates in the SAE, which allows the algorithms to detect EB corners better.

### 3.4. 3D Reprojection Error Performance

**Figure 5:**
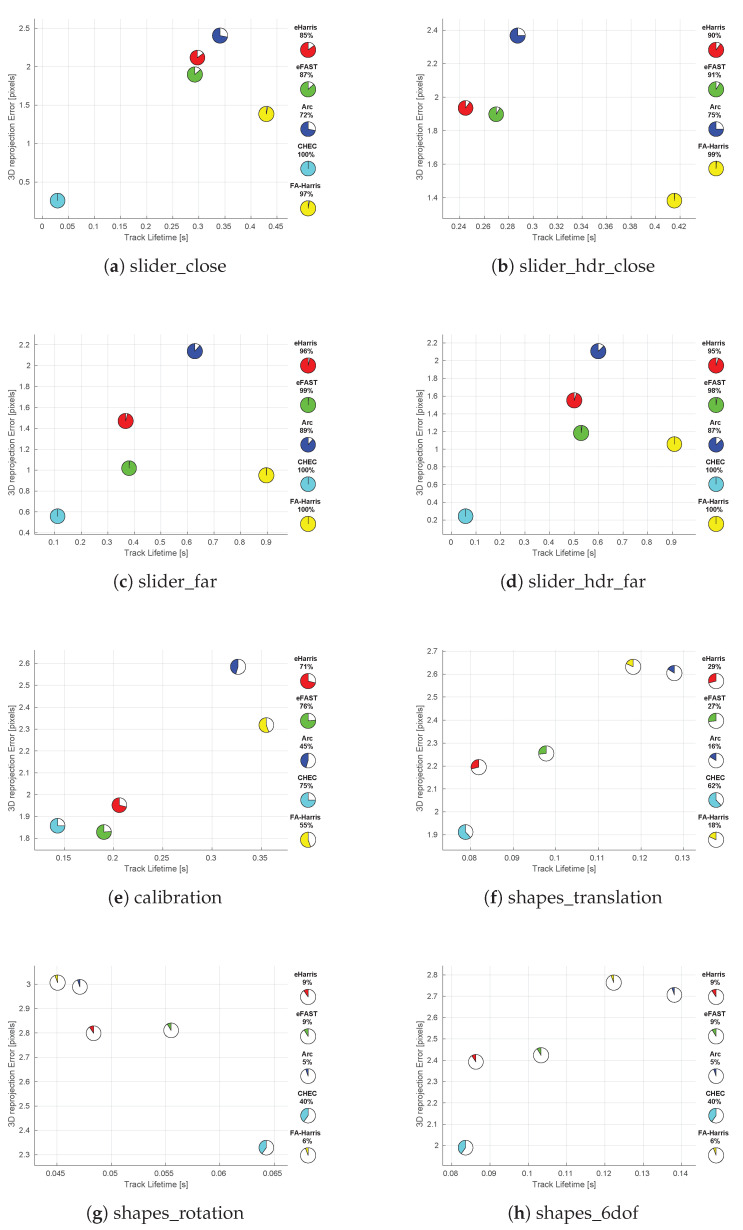
Average 3D reprojection error versus the average lifetime of feature tracks resulting from EB corner detector outputs for datasets: (**a**) slider_close, (**b**) slider_hdr_close, (**c**) slider_far, (**d**) slider_hdr_far, (**e**) calibration, (**f**) shapes_translation, (**g**) shapes_rotation, (**h**) shapes_6dof.

Comparing EB corner detectors only with intensity corners provides a better understanding of their similarities. However, this limits the evaluation to the operational region of frame-based cameras such as slow, favourable illumination conditions. Therefore, this part of the evaluation investigates their performance from the speed and illumination perspective. Figure 5 shows the 3D reprojection error performance of each EB detector for such varying scenes. Each EB corner detector is colour coded, and the percentage of valid tracks is shown with a pie chart and given within, as well as at the side legend of the figure.

Over all the datasets presented, the shortest average feature track times of all EB detectors are observed for the shapes_rotation dataset Figure 5g. This suggests that EB corner detectors are affected by rotational motions. Interestingly, CHEC corners have the longest feature track lifetime in the shapes_rotation dataset compared to other EB corner detectors, despite showing the worst lifetime performance in all other datasets. Considering the fact that CHEC somehow establishes a short-term spatiotemporal memory in the vicinity of an event spike, the exploitation of such memory could be an interesting avenue to explore a rotation invariant EB corner detector.

The calibration dataset presents a slower trajectory compared to the shapes dataset, while both last around 60 s and consist of geometrical patterns. As can be seen clearly in Figure 5e, the percentage of valid tracks and the track lifetime of all EB detectors are higher and longer than their performance on the shapes dataset in Figure 5f–h.

The slider_hdr datasets observe the same scene as the slider datasets, but the second half of their trajectory is affected by the harsh illumination conditions. For EB corner detectors, except CHEC, we do not observe any performance variation between these two types of datasets. In the slider_hdr_close dataset, CHEC corners were not sufficient to form feature tracks. This could be related to the selection of a constant Harris corner threshold for the entire dataset and searching at a single scale. Even though CHEC shows a better reprojection error for the slider_hdr_far dataset compared to slider_far, the feature tracks are significantly shorter than for the other EB corner detectors; therefore, this result could be due to the fortunate small parallax, and it is hard to deduce anything reasonable.

### 3.5. Computational Performance

All experiments were run on a single core of an Intel Core i7-4702MQ CPU at 2.20 GHz using a single threaded C++ implementation. Table 4 shows the average process duration for a single event and the respective maximum event rate in millions of events per second for each detector. For comparison, we also provide the real-time performance of each EB corner detector for the shapes_translation and boxes_translation datasets. Except eHarris, all EB corner detectors could achieve real-time performance for the shapes_translation dataset. However, the significant event rate increase for the boxes_translation dataset cannot be handled by EB corner detectors, except the Arc detector, which is implemented to perform over a reduced number of events. Moreover, we also observe that eHarris performs worse than FA-Harris, which simply implements eHarris on refined events. These results suggest that implementing pre-processing to reduce the number of events for computations with a higher complexity can improve the real-time performance of EB corner algorithms.

## 4. Discussion

Our analysis suggests the that reduction rates of the selected EB corner detectors are more affected by the scene structure than by the trajectory profile. When the illumination of a scene increases significantly without changing anything else, EB cameras stream less events; however, this does not change the reduction rate of EB corner detectors. This decrease in the number of event is due to the logarithmic sensitivity of EB detectors. Since the reduction rate is not affected in such a condition, the number of EB corners provided will also decrease. Therefore, higher level algorithms should be designed to adapt to such a change. Overall, Arc shows the lowest reduction rate, while CHEC and FA-Harris show the highest.

Generally speaking, the TPR performances of EB corner detectors are also found to be more highly correlated with the scene structure than the trajectory profile. The CHEC detector shows significant degradation in TPR performance with decreasing speed, which is related to the low update rate of the internal gradients. FA-Harris shows the worst performance among all EB corner detectors, except for the slider dataset, even though FA-Harris claims to achieve better performance by combining the strengths of eHarris and Arc. The overall performance of eHarris is found to be good. Even though this could be an indication of a close relationship between the SAE and intensity frames, such reasoning requires further analysis in order to be justified. We also found that the accumulation of events that is required for the computations of all EB corner detectors creates a delay between the start of the acquisition and nominal detection performance.

In terms of reprojection error, we found that the selected EB corner detectors were not rotation invariant. However, CHEC had a comparatively better response to this variation, suggesting that the exploitation of short-term spatiotemporal memory in the vicinity of event spikes could be an avenue to investigate. Increasing trajectory speeds were also shown to degrade the quality of the EB corners. Harsh illumination conditions did not affect the quality of the detected EB corners.

Arc shows the best performance among all EB corner detectors in terms of computational time. This is related to the prefiltering of events before performing time-consuming corner detection calculations. This is the main difference between the Arc and eFAST algorithms in terms of computational complexity.

Our analysis concludes that the current usage of prefiltering has a positive impact on algorithm computational complexity. Scene structure and speed show more significant effects on EB corner detectors than the trajectory profile (i.e., rotation, translation). The performances of current state-of-the-art solutions are robust to harsh illumination conditions. Arc is found to be a good compromise for real-time constraints and satisfactory accuracy. However, Arc still suffers from rotational motion and requires some time for nominal operational performance after the start of the acquisition. Nevertheless, none of these solutions are truly event-based: they all rely on some sort of spatiotemporal accumulation for their computations. In order to tap into the real potential of EB cameras, a true EB corner detection and tracking algorithm is needed.

## 5. Conclusions

This paper provides two main contributions. First, our study provides the first step in a more rigorous comparison of EB corner detection algorithms through our unified EB corner evaluation procedure, which could become the gold standard. This is ever more important since EB processing has now reached a new maturity: there are increasingly numerous databases and a fair number of existing EB corner detection algorithms to compare. Any new proposition of an EB algorithm can and should be evaluated rigorously.

Second, by applying our unified EB corner detection evaluation protocol to the most well-known EB corner detectors, we can highlight that current EB corner detectors seem to be reasonably robust to illumination conditions, but their performance still depends on the scene structure and the present trajectory. Above all, theses solutions require the accumulation of events in order to perform their calculations. Thus, to achieve a real EB corner detection and tracking algorithm, these issues still need to be addressed.

## Figures and Tables

**Table 1:** Event-Based (EB) corner detector algorithms’ properties and their respective paper’s evaluation criteria. eHarris, event-based Harris corner detector; eFAST, event-based FAST corner detector; CHEC, Continuous Harris Event Corner detector; FA-Harris, Fast and Asynchronous Harris corner detector; SITC, Speed Invariant Time surface corner detector; HDR, High Dynamic Range.

	localPlane	eHarris	eFAST	Arc	CHEC	FA-Harris	SITC
	[7]	[8]	[9]	[10]	[11]	[13]	[12]
**Algorithm Properties**						
Uses surface of active events	****✓****	**✓**	**✓**	**✓**	****✗****	**✓**	**✗**
Event-by-event processing	**✓**	**✓**	**✓**	**✓**	**✓** *	**✓**	**✓**
Adaptation of an image-based technique	**✗**	**✓**	**✓**	**✓**	**✓**	**✓**	**✓**
**Evaluation Criteria**						
Quantitative testing	**✓**	**✓**	**✓**	**✓**	**✗**	**✓**	**✓**
Tested on a public dataset	**✗**	**✗**	**✓**	**✓**	**✓**	**✓**	**✓** **
Testing on scenes ≥60 s	**✗**	**✗**	**✓**	Partial	N/A	Partial	**✓**
Tested on fast moving scenes	**✓**	**✓**	**✓**	Partial	Partial	Partial	**✓**
Tested on HDR scenes	**✗**	**✗**	**✗**	**✗**	**✗**	**✗**	**✓**
Manually labelled ground truth	**✓**	**✓**	**✗**	**✗**	**✗**	**✗**	**✗**
Uses camera trajectory	**✓**	**✓**	**✗**	Partial	N/A	Partial	**✓**
Ground truth requires on intensity frames	**✗**	**✗**	**✗**	**✓**	N/A	**✓**	**✗**

* With degradation of the performance; ** the proposed public ATIS dataset does not include the pixel intensity values used for the algorithm training.

**Table 2:** Reduction rate of EB corner detectors per algorithm for each scene. For readability, the highest value for each row is highlighted in bold; this does not necessarily correspond to the best algorithm, as this metric should be read in conjunction with the other results.

Scene	Total # of Events	eHarris (%)	eFAST (%)	Arc (%)	CHEC (%)	FA-Harris (%)
calibration	21,340,629	92.1	95.3	92.8	95.6	**98.3**
shapes_translation	17,363,976	91.7	87.8	88.1	**98.3**	95.6
shapes_rotation	23,126,288	92.7	88.9	87.7	**99.1**	95.4
shapes_6dof	17,962,477	90.6	87.0	88.3	**97.8**	95.7
dynamic_translation	35,809,924	95.3	96.7	92.7	96.3	**98.7**
dynamic_rotation	71,324,510	95.1	96.4	91.7	**98.2**	98.0
dynamic_6dof	57,174,637	95.4	96.4	92.2	97.6	**98.3**
poster_translation	100,033,286	92.3	95.8	90.9	**98.3**	97.8
poster_rotation	169,350,136	92.6	95.7	89.9	97.3	**97.4**
poster_6dof	133,464,530	92.4	95.6	90.2	**97.5**	**97.5**
hdr_poster	102,910,720	93.5	96.2	92.4	98.2	**98.3**
boxes_translation	112,388,307	92.4	96.7	92.2	**99.1**	97.9
boxes_rotation	185,688,947	92.1	96.7	91.9	97.5	**97.7**
boxes_6dof	133,085,511	92.7	96.8	92.2	**98.2**	98.0
slider_close	4,032,668	92.8	92.7	89.8	**100.0**	97.6
slider_far	3,442,683	91.7	95.8	92.8	**99.9**	98.3
slider_hdr_close	3,337,787	95.4	93.8	90.4	**100.0**	98.4
slider_hdr_far	2,509,582	91.7	94.2	90.9	**100.0**	97.8

**Table 3:** True Positive Rate (TPR) of EB corner detectors for given scenes. For each row, the best score per radius value is highlighted in bold.

Scene	eHarris			eFAST			Arc			CHEC			FA-Harris		
Radius (pix)	1.5	3.5	5	1.5	3.5	5	1.5	3.5	5	1.5	3.5	5	1.5	3.5	5
calibration	**20.4**	**16.5**	**13.0**	16.0	12.7	9.8	11.7	11.0	9.5	17.9	14.1	11.4	3.6	3.1	2.4
shapes_translation	15.4	13.1	10.5	**22.6**	**19.3**	**15.4**	13.6	11.7	9.5	7.8	7.0	6.3	5.7	4.8	3.6
shapes_rotation	16.6	14.7	11.4	**23.0**	**20.1**	**15.8**	14.6	12.8	10.3	7.6	6.7	5.9	6.2	5.5	4.0
shapes_6dof	15.8	13.5	11.0	**21.5**	**18.9**	**15.4**	11.4	10.6	9.0	12.4	11.0	10.0	4.3	3.7	2.9
dynamic_translation	**11.1**	8.1	7.0	6.7	4.8	4.1	10.1	**9.2**	**8.6**	7.3	7.0	7.1	2.5	1.8	1.5
dynamic_rotation	10.4	9.2	8.4	6.5	5.8	5.4	**11.3**	**11.5**	**11.3**	3.9	3.8	3.8	2.9	2.7	2.4
dynamic_6dof	**10.0**	7.9	6.6	6.6	4.8	4.0	9.8	**9.0**	**8.4**	7.7	7.1	6.8	2.2	1.6	1.3
poster_translation	**11.1**	**10.0**	**9.0**	4.1	4.1	3.7	6.3	6.4	6.4	3.7	3.9	3.8	1.6	1.5	1.4
poster_rotation	**12.8**	**11.6**	**10.1**	5.2	5.1	4.3	7.5	7.3	6.8	2.7	3.2	3.0	1.9	1.9	1.6
poster_6dof	**12.5**	**11.0**	**9.8**	4.7	4.5	4.0	6.8	6.6	6.3	4.1	4.6	4.5	1.4	1.3	1.2
boxes_transl.	**12.5**	**10.4**	**8.9**	4.1	3.6	3.2	7.1	6.5	6.1	2.3	2.0	2.0	2.1	1.7	1.4
boxes_rotation	**9.5**	**9.0**	**8.3**	3.1	2.8	2.8	6.0	5.7	5.8	1.2	1.2	1.1	1.7	1.5	1.4
boxes_6dof	**11.1**	**9.1**	**8.0**	3.6	3.0	2.7	6.6	6.2	5.8	1.4	1.2	1.1	1.7	1.4	1.2
slider_close	**16.7**	**16.2**	14.5	12.4	14.9	13.2	14.5	16.1	**15.2**	0.4	0.1	0.1	4.8	5.5	5.0
slider_far	**16.1**	**14.6**	**12.1**	5.3	6.2	5.2	10.5	9.3	8.3	0.2	0.1	0.1	2.7	2.4	2.0

**Table 4:** Computational performance of event corner detectors for given scenes.

Detector	Time Per Event	Max. Event Rate	Real-Time Performance	
	(s/event)	(Mevents/s)	shapes_trans.	boxes_trans.
eHarris	6.93	0.14	**✗**	**✗**
eFAST	1.13	0.88	**✓**	**✗**
Arc	**0.22**	**4.65**	**✓**	**✓**
CHEC	1.90	0.53	**✓**	**✗**
FA-Harris	1.11	0.90	**✓**	**✗**

## Data Availability

Data available in a publicly accessible repository that does not issue DOIs. Publicly available datasets were analyzed in this study. This data can be found here: http://rpg.ifi.uzh.ch/davis_data.html.
